# Real-world management and long-term outcomes in adolescent, young adult, and adult medulloblastoma: Experience from a monocentric series with multimodal and targeted approaches

**DOI:** 10.1093/nop/npaf110

**Published:** 2025-10-24

**Authors:** Alberto Bosio, Marta Maccari, Marta Padovan, Mario Caccese, Maital Bolshinsky, Antonella Galiano, Francesco Cavallin, Luisa Bellu, Francesco Pasqualetti, Alessandro Parisi, Giovanni Librizzi, Giovanna Pintacuda, Alessandro Salvalaggio, Marco Zoccarato, Tamara Ius, Francesco Volpin, Luca Denaro, Sara Lonardi, Giuseppe Lombardi

**Affiliations:** Department of Surgery, Oncology and Gastroenterology, University of Padova: Università degli Studi di Padova, Padova, Italy; Department of Surgery, Oncology and Gastroenterology, University of Padova: Università degli Studi di Padova, Padova, Italy; Medical Oncology 1, Veneto Institute of Oncology IOV—IRCCS, Padova, Italy; Medical Oncology 1, Veneto Institute of Oncology IOV—IRCCS, Padova, Italy; Medical Oncology 1, Veneto Institute of Oncology IOV—IRCCS, Padova, Italy; Medical Oncology 1, Veneto Institute of Oncology IOV—IRCCS, Padova, Italy; Medical Oncology 1, Veneto Institute of Oncology IOV—IRCCS, Padova, Italy; Independent Statistician, Solagna, Italy; Radiotherapy Unit, IOV-IRCCS Veneto Institute of Oncology, Padova, Italy; Department of Surgery, Oncology and Gastroenterology, University of Padova: Università degli Studi di Padova, Padova, Italy; Radiotherapy Unit, IOV-IRCCS Veneto Institute of Oncology, Padova, Italy; Radiotherapy Unit, IOV-IRCCS Veneto Institute of Oncology, Padova, Italy; Padova Neuroscience Center (PNC), University of Padova: Università degli Studi di Padova, Padova, Italy; Neuroradiology Unit, Padova University Hospital, Padova, Italy; Radiology Unit, Veneto Institute of Oncology IOV-IRCCS, Padova, Italy; Padova Neuroscience Center (PNC), University of Padova: Università degli Studi di Padova, Padova, Italy; Department of Neuroscience, University of Padova: Università degli Studi di Padova, Padova, Italy; Neurology Unit O.S.A, Azienda Ospedale-Università di Padova, Padova, Italy; Academic Neurosurgery, Department of Neurosciences, University of Padova: Università degli Studi di Padova, Padova, Italy; Division of Neurosurgery, Azienda Ospedaliera Università di Padova, Padova, Italy; Academic Neurosurgery, Department of Neurosciences, University of Padova: Università degli Studi di Padova, Padova, Italy; Medical Oncology 1, Veneto Institute of Oncology IOV—IRCCS, Padova, Italy; Medical Oncology 1, Veneto Institute of Oncology IOV—IRCCS, Padova, Italy

**Keywords:** AYA/Adults, de-escalation, medulloblastoma, targeted therapy

## Abstract

**Abstract:**

Background Medulloblastoma (MB) is an exceedingly rare malignancy in adolescents and young adults (AYA) and adults. This study aims to describe clinical characteristics, treatments, including the use of targeted therapy, outcomes, and long-term sequelae in a single-center cohort of AYA/adult MB patients.

**Methods:**

We retrospectively analyzed MB patients aged ≥16 years treated at Veneto Institute of Oncology, Padua, Italy, between 2011 and 2025. Clinical, molecular, and treatment data were collected. Treatment response was assessed using RAPNO criteria, and treatment-related toxicity according to CTCAE v5.0. Survival outcomes were estimated using the Kaplan-Meier method.

**Results:**

We included 29 patients. At diagnosis, 86% were symptomatic, 55% had an ECOG performance status (PS) of 0, and desmoplastic/nodular was the most frequent histology (48%). Molecular subgrouping was available in 45%, with SHH activation as the most common subgroup (31%, 9/29 patients). All patients underwent craniospinal radiotherapy; adjuvant chemotherapy regimens included cisplatin-etoposide±cyclophosphamide (62%), cisplatin-lomustine±vincristine (21%), and intensive pediatric regimens (10%), while 2 patients (7%) did not receive chemotherapy due to comorbidities. First-line complete response was achieved in 65%. Overall, 38% experienced progression, and 24% died. Three patients received vismodegib at recurrence. Three-year PFS and OS rates were 78% and 92%. Stratified analyses indicated worse outcomes in patients with high-risk classification, subtotal resection, and lower baseline ECOG PS. Grade 3-4 toxicity occurred in 30%. Long-term complications included neurocognitive impairment (21%) and radiotherapy-induced secondary malignancies (10%).

**Conclusions:**

Long-term survivorship is often achievable in AYA/adult MB, though late toxicities remain a concern. Molecular profiling supports risk stratification and therapy personalization. Long-term toxicities highlight the need for de-intensified strategies.

Key points

- Long-term survivorship is often achievable with a multimodal approach.
- AYA/adult medulloblastoma survivors face significant neurocognitive late effects.
- Findings support de-escalation strategies to balance efficacy and survivorship.

Medulloblastoma (MB) is a highly malignant embryonal tumor of the posterior fossa and represents the most common malignant brain tumor in children.^1^ Although rare in adults, it accounts for a significant proportion of posterior fossa tumors in the adolescent and young adult (AYA) population, typically defined as patients between 15 and 39 years old at the time of their initial cancer diagnosis.^2^ The incidence of MB in adults has been estimated to be less than 1 case per million per year, with a distinct epidemiological and molecular profile compared to pediatric cases.^3^^,^^4^ In the AYA population, the tumor exhibits overlapping biological features with both pediatric and adult subgroups, necessitating age-tailored therapeutic approaches.^5^ Due to its rarity in older patients, current treatment strategies are largely extrapolated from pediatric protocols, although recent studies have underscored the need for distinct management strategies in adults.^6–8^

The 2021 WHO CNS tumor classification highlights the relevance of molecular subgrouping in MB, which includes WNT-activated, SHH-activated (with TP53-wildtype or mutant), and non-WNT/non-SHH subgroups.^9^ SHH-activated MB is particularly common in adult patients, which has justified the exploration and clinical use of SMO (smoothened) inhibitors such as vismodegib and sonidegib.^10^^,^^11^ Despite promising preclinical data, real-world outcomes in adults remain underreported.

This retrospective study presents a comprehensive analysis of AYA/adult MB patients treated over a 13-year period at a single academic Center. We report clinical features, molecular characteristics, treatment strategies including the use of targeted therapy, and both survival outcomes and long-term toxicities. By comparing our findings with available literature, we aim to inform clinical practice and future research directions.

Importance of the study

Medulloblastoma in adolescents and young adults (AYA) and adults is rare and underrepresented in clinical research, with treatment strategies often extrapolated from pediatric protocols. This single-center retrospective study offers one of the largest real-world analyses of AYA/adult medulloblastoma treated with multimodal approaches, integrating molecular profiling and targeted therapy. The results show that long-term survivorship is achievable, although late toxicities—particularly neurocognitive impairment and secondary malignancies—remain a major concern. Importantly, it supports the need for de-escalation strategies in selected patients to reduce long-term sequelae without compromising efficacy. These findings contribute evidence for refining current management and may inform future clinical decision-making and research priorities.

## Methods

We conducted a retrospective review of patients aged greater than or equal to 16 years diagnosed with histologically confirmed MB treated at Veneto Institute of Oncology, Padua, Italy between November 2011 and February 2025. Relevant data were extracted from electronic medical records and included patients’ demographics, baseline clinical features, treatment details, and outcomes.

Patients still alive at the time of this study provided informed consent to participate in the ANIMA trial (Analysis of clinical, radiological and Molecular Alterations in adult primary central nervous system tumors, and their impact on treatment decision making and patients’ outcome; IOV-2024-ANIMA). Patients who had died prior to the study period were included as part of the retrospective cohort, in accordance with ethical standards and privacy regulations. All data concerning enrolled patients were anonymized and recorded in a dedicated, secure database accessible only to authorized study personnel.

Risk stratification followed modified pediatric criteria based on Chang M-stage, extent of resection, histology and molecular subgroup. The Chang staging system classifies MB according to tumor size (T-stage) and extent of metastatic spread (M-stage), where M0 indicates no evidence of metastases, M1 denotes microscopic tumor cells in cerebrospinal fluid, and M2-M4 indicate increasing degrees of macroscopic metastatic disease within the neuraxis or extraneurally (see Figure S1, Supplements).

Patients were classified into two risk categories by integrating clinical, histopathological, and molecular features. Intermediate-risk was defined as non-metastatic disease (M0), gross total or near-total resection (≤1.5 cm² residual tumor), and absence of high-risk histological or molecular features. High-risk included metastatic disease (M1-M4), residual tumor >1.5 cm², large cell/anaplastic histology, MYC or MYCN amplification, or TP53-mutant SHH tumors. Molecular classification was performed when tissue was available, using immunohistochemistry and/or DNA methylation profiling, and SHH activation was defined by canonical pathway markers. Response to therapy was assessed according to the Response Assessment in Pediatric Neuro-Oncology (RAPNO) criteria (these criteria standardize response evaluation in MB and other leptomeningeal seeding tumors. They incorporate MRI of the brain and spine, neurologic examination, and CSF cytology; see Figure S2, supplements), while adverse events were graded using the Common Terminology Criteria for Adverse Events (CTCAE) version 5.0. Statistical analysis was performed using R 4.4 (R Foundation for Statistical Computing, Vienna, Austria). Categorical data were summarized as number and percentage, and continuous data as median and interquartile range (IQR). Survival curves were calculated using the Kaplan–Meier method.

## Results

### Patient Characteristics

Twenty-nine patients were evaluated. Patients’ characteristics at baseline are summarized in Table 1. Median age at diagnosis was 34 years (IQR 22-41), with a slight female predominance (55.5%). At presentation, 86% were symptomatic, with headaches and cerebellar signs being the most frequent symptoms. ECOG PS was 0 in 55%. Histology revealed desmoplastic/nodular subtype in 48% of patients, followed by classic (31%), and anaplastic (3.5%). Five patients (17.2%) were classified as MB not otherwise specified, since further information about histology were not available. Eighteen (62.1%) patients had a M0 stage according to Chang-classification and 10 (34.5%) did not undergo diagnostic lumbar puncture due to a perceived high risk of tonsillar herniation. Molecular classification was available in 13 cases (44.8%), with SHH activation in9 out of 29 patients (31%), WNT in 1 out of 29 patients (3.5%), and non-WNT/non-SHH in 3 out of 29 patients (10.3%).

**Table 1.** Patients’ baseline characteristics

All MB patients	*N* = 29
Age at diagnosis, years	34 (IQR 22-41)
Sex	
Male	13 (44.5%)
Female	16 (55.5%)
ECOG PS at diagnosis	
0	16 (55.2%)
1	9 (31.0%)
2	3 (10.3%)
3	1 (3.5%)
Symptomatic patients^a^	25 (86.2%)
Chang M-stage^b^	
M0	18 (62.1%)
M1	0 (0%)
M2	3 (10.3%)
Unknown	8 (27.6%)
Risk class	
Intermediate risk	14/25 (60.0%)
High risk	10/25 (40.0%)
Tumor size (maximum diameter), mm	40 (IQR 20-46)
Histology	
Anaplastic	1 (3.5%)
Classic	9 (31.0%)
Desmoplastic/nodular	14 (48.3%)
MB NOS	5 (17.2%)
Molecular subgroup	
Non WNT, non SHH	3 (10.3%)
SHH activated	9 (31.0%)
WNT activated	1 (3.5%)
Not available	16 (55.2%)
Lumbar puncture	19 (65.5%)
CSF positive	1/19 (5.3%)
EOR	
Subtotal	16 (55.2%)
Gross total	13 (44.8%)

Abbreviations: CSF, Cerebrospinal Fluid; ECOG PS, Eastern Cooperative Oncology Group performance status; EOR, extent of resection; NOS, not otherwise specified; SHH, Sonic Hedgehog.

a Symptoms included ataxia (*n* = 6), dysarthria (*n* = 1), headache (*n* = 18), seizures (*n* = 1), paresthesia (*n* = 1), dizziness (*n* = 10), diplopia (*n* = 3), phosphenes/subjective visual sensations (*n* = 1), vomiting (*n* = 3), hypoacusis (*n* = 1), hemisyndrome (*n* = 1), and tinnitus (*n* = 1).

b Chang M-stage classification: M0, no metastases; M1, microscopic metastases in cerebrospinal fluid; M2, gross nodular seeding in cerebellar/cerebral subarachnoid space or in the third/fourth ventricles; M3, gross nodular seeding in spinal subarachnoid space; M4, extraneural metastases.

First-line treatment data are reported in Table S1. All patients underwent photon-based craniospinal irradiation (median dose of 53.6 Gray) and 22 (75.9%) of them received focal boost on tumor bed.

Adjuvant chemotherapy was administered to 27 patients (93%). The most frequent regimen was cisplatin-etoposide with or without cyclophosphamide (*N* = 18, 62%), followed by cisplatin-lomustine with or without vincristine (*N* = 6, 21%) and intensive pediatric regimens (*N* = 3, 10%), comprising cyclophosphamide, vincristine, methotrexate, carboplatin, and etoposide, plus intraventricular methotrexate (36 single doses; 2 mg/dose). Two patients (7%) were deemed unfit for chemotherapy due to comorbidities. The choice of the chemotherapeutic regimen was left to the discretion of the treating physician.

### Survival Outcomes

**Figure 1:**
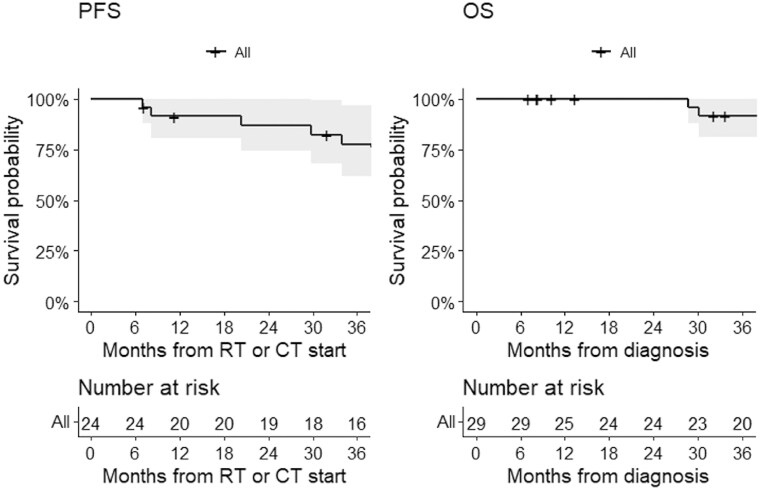
Progression-free survival (left) and overall survival (right) in patients who were diagnosed with MB between November 2011 and June 2024. Progression-free survival could not be calculated in 5 patients due to missing information.

**Figure 2:**
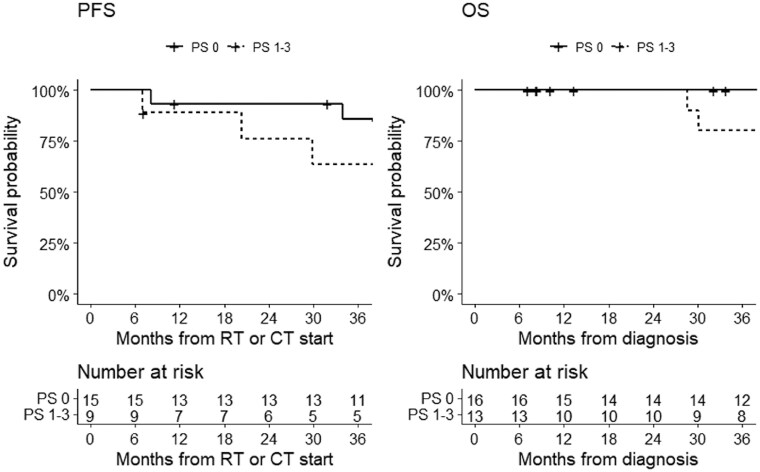
Progression-free survival (left) and overall survival (right) in MB patients with performance status 0 and those with performance status 1-3.

At a median follow-up of 71 months (IQR 32-112), 38% of patients had disease progression and 24% had died. The 3-year PFS and OS were 78% and 92%, respectively. Progression-free survival at 1-2-3 years was 92-87-78% and OS at 1-2-3 years was 96-96-92% (Figure 1). Stratifying by ECOG PS at diagnosis, the 3-year PFS was 85% in patients with ECOG PS 0 and 63% in those with ECOG PS 1-3; 3-year OS was 90% in patients with ECOG PS 0 and 80% in those with ECOG PS 1-3 (Figure 2).

**Figure 3:**
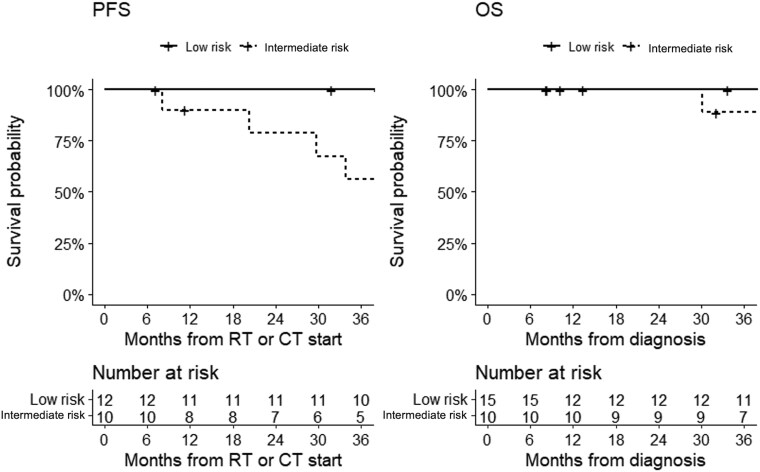
Progression-free survival (left) and overall survival (right) in intermediate-risk medulloblastoma patients and high-risk medulloblastoma patients. Progression-free survival (PFS) analysis was performed in 22 patients with complete follow-up data, whereas overall survival (OS) analysis included 25 patients in this subgroup analysis.

**Figure 4:**
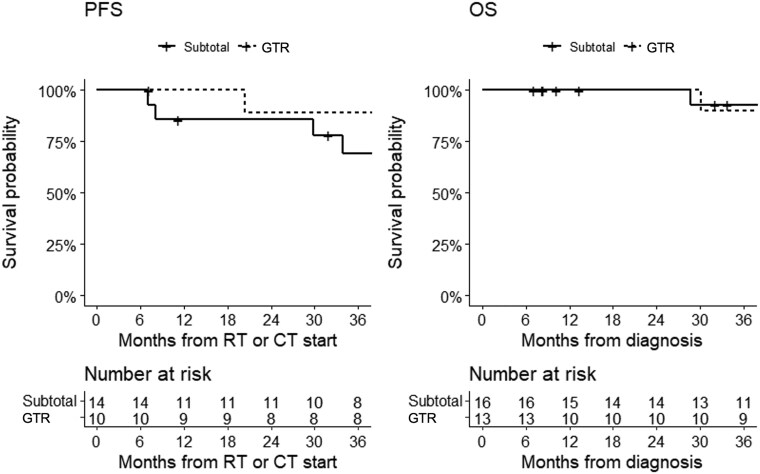
Progression-free survival (left) and overall survival (right) in MB patients who underwent subtotal resection and those who underwent gross total resection. Progression-free survival could not be calculated in 5 patients due to missing information. GTR: gross total resection.

**Figure 5:**
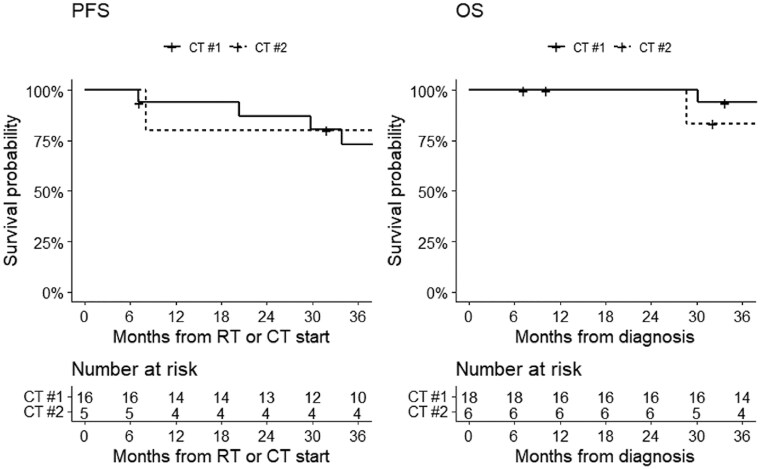
Progression-free survival (left) and overall survival (right) in MB patients treated with cisplatin-etoposide with or without cyclophosphamide (CT #1) and those treated with cisplatin-lomustine with or without vincristine (CT #2). Progression-free survival could not be calculated in 5 patients due to missing information.

Stratifying by risk status, the 3-year PFS was 88% in intermediate-risk patients and 56% in high-risk; the 3-year OS was 90% in intermediate-risk patients and 88% in high-risk patients (Figure 3). Stratifying by resection, 3-year PFS was 69% in patients who underwent subtotal resection and 88% in those who underwent gross total resection; 3-year OS was 93% in patients who underwent subtotal resection and 90% in those who underwent gross resection (Figure 4). Survival analysis according to main chemotherapy schemes showed a 3-year PFS of 73% in patients treated with cisplatin-etoposide with or without cyclophosphamide and 76% in those treated with cisplatin-lomustine with or without vincristine; 3-year OS of 93% in patients treated with cisplatin-etoposide tabwith or without cyclophosphamide and 83% in those treated with cisplatin-lomustine with or without vincristine (Figure 5).

### Radiological Response

The radiological best response to first-line treatment was complete response in 19 patients (65.5%), partial response in five patients (17.2%), and stable disease in one patient (3.5%). Radiological assessment was not available for four patients (13.8%). No patient exhibited progressive disease (PD) as a best-response to first-line treatment.

### Toxicity

Twenty-three were evaluable for first-line chemotherapy-related safety. Seven of them (30.4%) experienced a grade 3-4 toxicity. Particularly, grade 3-4 neutropenia was the most frequent adverse event (*N* = 7), with one patient experiencing febrile G4 neutropenia which required hospitalization, followed by grade 3-4 anemia (*N* = 2) and thrombocytopenia (*N* = 1).

### Use of Vismodegib

Overall, 3 out of 29 patients (10.3%) received second-line treatment with vismodegib. One patient (M0 stage, high-risk) received 3 cycles without toxicity, had progressive disease at 3 months from vismodegib start and died at 30 months from diagnosis. One patient (M2 stage, high-risk) received 5 cycles without toxicity, progressed at 4 months and has been followed for 12 months since disease progression. One patient (M0 stage, high-risk) received 11 cycles, developed G1 toxicity (fatigue, muscular spasms), and did not experience progressive disease until now.

### Long-Term Sequelae

Long-term complications were observed in nine patients (31%), including cataracts (*N* = 5), radiation-induced tumors (*N* = 3, one thyroid carcinoma, one meningioma, one dorsal pleomorphic sarcoma; median time to diagnosis 8 years, range 6-10 years), and cisplatin-related paresthesia (*N* = 1). Neurocognitive impairment affected six patients (21%), presenting as psychiatric disorders (*N* = 4, including anxiety, major depressive symptoms, emotional lability and social withdrawal), short-term memory deficits (*N* = 1), and bilateral sensorineural hearing loss (*N* = 1).

## Discussion

This study underscores the clinical heterogeneity of AYA/adult MB and the feasibility of long-term disease control using a tailored multimodal approach. Our 3-year OS of 92% is encouraging and aligns with recent retrospective series.^12–14^ Predictably, inferior outcomes were found in patients with high-risk disease, poor performance status, and subtotal resection.

Surgical resection continues to be a fundamental component of the management of MB, with the primary objective of minimizing neurological sequelae and accomplishing the safest tumor removal possible. The prognostic benefit of increased extent of resection is attenuated when molecular subgroup affiliation is considered, indicating that biology may be more important than the extent of resection in determining long-term outcomes.^4^ However, recent evidence suggests that the survival advantage of gross total resection (GTR) over near-total resection (NTR) may not be significant, particularly in SHH-activated and WNT subgroups, despite the traditional association between GTR and enhanced survival, particularly in historical cohorts.^4^^,^^15^ Therefore, while the standard of care should be the maximal safe resection, the surgical removal of small residual portions of the tumor is not advisable when the risk of neurological morbidity is high.

Our cohort included two main chemotherapy backbones: cisplatin-etoposide (with or without cyclophosphamide) and cisplatin-lomustine (with or without vincristine), both extrapolated from pediatric protocols but variably used in adult practice. The regimen combining cisplatin and lomustine appeared to yield a longer progression-free survival compared to cisplatin and etoposide, a finding that is likely influenced by the smaller number of patients treated with the former and thus underpowered for definitive comparisons, rather than by a true difference in intrinsic efficacy. This aligns with data from the prospective NOA‑07 trial in adults, which analyzed the feasibility of the cisplatin‑lomustine-vincristine combination highlighting the need for age-adjusted dosing strategies given the notable hematologic and neurologic toxicities observed.^8^ Indeed, we preferred to eliminate vincristine from the treatment due to the high hematological and neurological toxicity in adults. Temozolomide has also shown activity in the recurrent setting, as demonstrated in the Italian multi-institutional phase II trial conducted in pediatric patients with MB, suggesting a potential role worth exploring in the AYA and adult population, particularly in those requiring less intensive, well-tolerated regimens.^16^ Our findings contribute real-world data on the use of vismodegib in adult SHH-MB. In our cohort, three patients received vismodegib, with variable efficacy. One patient achieved prolonged disease control, while two others experienced early progression despite the absence of significant toxicity. This variability reflects findings from prior studies, where vismodegib has shown heterogeneous responses, particularly in patients with acquired resistance mechanisms such as SMO mutations or downstream pathway activation. For instance, Robinson et al. and Kool et al. have reported objective response rates in the range of 10%-20%, often transient, with better outcomes in patients without TP53 mutations or MYCN amplification.^10^^,^^11^ Compared to our data, these studies often included patients with heavier pretreatment and more aggressive disease phenotypes. Our patient who benefited most from vismodegib had favorable clinical parameters and a longer disease-free interval before recurrence, suggesting the timing and patient selection are crucial for optimizing benefit from SMO inhibitors. Moreover, initiating vismodegib earlier in the treatment course may help circumvent downstream mutations within the Sonic Hedgehog pathwaysuch as those affecting SUFU or GLI—SUFU (Suppressor of Fused) and GLI (Glioma-associated oncogene homolog)that have been implicated in resistance to SMO inhibitors, as suggested by several case reports.^17^^,^^18^ The tolerability profile of vismodegib observed, limited to grade 1 fatigue and muscle cramps, is consistent with prior trials and supports its use in carefully selected SHH-activated cases.^19^

Regarding treatment-related toxicities, our data confirm the substantial burden associated with current standard therapies. Acute toxicity affected the majority of patients, with nearly one-third experiencing grade 3-4 events. These rates are in line with previous adult MB series and underscore the challenges of adapting pediatric-inspired regimens to an older population often with lower physiological reserve.^7^^,^^8^^,^^14^

Long-term sequelae were also prevalent in our cohort and likely underestimated due to retrospective nature and non-systematic reporting. Neurocognitive dysfunction was noted in around one out of five patients, a finding consistent with prior observational studies, especially those employing neuropsychological testing.^20^ Radiation-induced malignancies occurred in around one out of ten patients, which was higher than the figures typically reported in pediatric series, possibly due to differences in radiation volume, older techniques with minor target conformality, or longer follow-up.^21^ Additionally, cataract formation and chemotherapy-induced neuropathy were observed, reinforcing the need for long-term monitoring and preventive strategies in survivorship care.

The rarity of adult MB, together with its biological heterogeneity, poses major challenges for the design and conduct of prospective clinical trials. Accrual is often slow even in multicenter or international settings, as exemplified by the early termination of the randomized EORTC 1634-BTG/NOA-23 trial, despite its strong rationale.^22^ Our study is one of the largest monocentric cohorts of AYA/adult MB patients with molecular data and detailed longitudinal follow-up. Similar findings have been reported in molecularly characterized adult MB series. Zhao et al. profiled 13 primary tumors and validated in 201 additional cases, showing that SHH-activated tumors (62%) predominate in adults, followed by Group 4 (28%) and WNT (10%), each associated with distinct clinical and prognostic features.^23^ Coltin et al. analyzed an international cohort of 191 adult MB using methylation and genomic profiling, confirming the predominance of SHH tumors and revealing subgroup-specific survival differences, with Group 4 showing the poorest outcomes.^24^ Moreover, the comprehensive documentation of both therapeutic responses and late effects provides relevant insights for clinical management and future trial design. Nevertheless, some limitations must be acknowledged. The retrospective design introduces inherent biases, including variability in treatment approaches over time and missing data, particularly in molecular characterization. More than half of the patients in our cohort lacked complete molecular characterization. This was mainly due to the use of archival formalin-fixed paraffin-embedded tissue, which in some cases was insufficient in quantity or quality for comprehensive molecular testing, and to the limited availability of molecular diagnostics in earlier years of the study period

Yet, the interpretation of complete response rates after first-line treatment is challenging in the context of craniospinal irradiation, as post-radiotherapy changes such as transient contrast enhancement, pseudoprogression, or radiation-induced edema may confound radiological assessment. These treatment-related effects, although less frequently reported in MB than in gliomas, could have led to an overestimation or misclassification of radiological responses in our cohort.

Moreover, we acknowledge that the small sample size and the clinical and molecular heterogeneity of adult MB in our series limit the generalizability of these results. Given the rarity of the disease, future research should aim to pool data from multiple institutions and cooperative groups to increase statistical power and refine prognostic and therapeutic recommendations for this patient population. Furthermore, the use of vismodegib was limited to a few cases, and the absence of detailed genomic profiling beyond SHH activation limits the interpretability of treatment outcomes.

## Conclusions

This study confirms the clinical and biological heterogeneity of AYA/adult MB and highlights the feasibility of achieving long-term disease control through a multimodal treatment approach. While survival outcomes are encouraging, the burden of late toxicities remains significant and calls for thoughtful survivorship care planning. The integration of molecular profiling into clinical decision-making, particularly in SHH-activated tumors, can improve risk stratification and potentially guide the adoption of targeted therapies such as vismodegib. However, the observed heterogeneity in response underscores the need for further biomarker-driven research. Future efforts should focus on prospective validation of molecular-based treatment strategies and the implementation of therapeutic de-intensification approaches to minimize long-term sequelae while maintaining disease control. Given the low incidence and heterogeneity of adult MB, large-scale prospective studies remain difficult to complete. Collaborative, multicenter approaches and pragmatic trial designs are essential to generate high-quality evidence in this rare disease setting.

## Supplementary Material

npaf110_Supplementary_Data

## Data Availability

The datasets generated during the current retrospective study are not publicly available.
