# Supplementary material for: Real-world management and long-term outcomes in adolescent, young adult, and adult medulloblastoma: Experience from a monocentric series with multimodal and targeted approaches
Source: Neurooncol Pract. 2025 Oct 24;13(2):385–93. doi: 10.1093/nop/npaf110 (PMC13153702; doi:10.1093/nop/npaf110)
Supplement: npaf110_Supplementary_Data [file npaf110_supplementary_data.docx]

**Supplementary**

| **Stage** | **Definition** |
| --- | --- |
| **T1** | Tumor < 3 cm in greatest diameter, confined to midline vermis, roof of the fourth ventricle, or cerebellar hemisphere. |
| **T2** | Tumor ≥ 3 cm and/or extending into one adjacent structure (e.g., floor of fourth ventricle, foramen of Luschka, or cerebellar peduncle). |
| **T3a** | Tumor infiltrating the midbrain. |
| **T3b** | Tumor invading the medulla oblongata or cervical spinal cord. |
| **T4** | Tumor extending into the aqueduct of Sylvius or upper cervical canal. |
| **M0** | No evidence of metastasis. |
| **M1** | Tumor cells present in cerebrospinal fluid. |
| **M2** | Gross nodular seeding in cerebellum, cerebral subarachnoid space, or in the third/lateral ventricles. |
| **M3** | Gross nodular seeding in spinal subarachnoid space. |
| **M4** | Extraneural metastases. |

Figure S1. Chang Staging System for medulloblastoma

| **Response Category** | **Definition** |
| --- | --- |
| **Complete Response (CR)** | Disappearance of all target lesions on MRI; no new lesions; no clinical evidence of disease; off corticosteroids (or on physiologic replacement doses only). |
| **Partial Response (PR)** | ≥ 50% decrease in the sum of the products of perpendicular diameters (SPD) of target lesions compared to baseline; no new lesions; stable/improved clinical status; stable/decreasing corticosteroids. |
| **Minor Response (MR)** | 25–49% decrease in SPD of target lesions compared to baseline; no new lesions; stable/improved clinical status; stable/decreasing corticosteroids. |
| **Stable Disease (SD)** | < 25% decrease or < 25% increase in SPD compared to baseline; no new lesions; stable clinical status. |
| **Progressive Disease (PD)** | ≥ 25% increase in SPD of target lesions compared to smallest recorded value (nadir) or appearance of new lesions; or clear clinical deterioration not attributable to other causes. |

Figure S2. RAPNO Criteria for medulloblastoma Response Assessment

Table S1. First-line treatment informations

| All medulloblastoma patients | N=29 |
| --- | --- |
| Radiotherapy | 29 (100%) |
| CSI  No  Yes  Unclear (therapy performed at other Center) | 1 (3.5%)  27 (93.0%)  1 (3.5%) |
| Focal boost  No  Yes  Unclear (therapy performed at other Center) | 3 (10.3%)  22 (75.9%)  4 (3.8%) |
| Total dose, Gy | 53.6 (53.6-53.6) |
| Chemotherapy  Cisplatin-etoposide$\pm$cyclophosphamide  Cisplatin-lomustine$\pm$vincristine  HIT SKK protocol  PNET5 protocol  None (due to comorbidities) | 18 (62.1%)  6 (20.7%)  2 (6.9%)  1 (3.5%)  2 (6.9%) |
| Median number of cycles | 4 (3-8) |
| Radiological response at first-line treatment  CR PR  SD  PD  Not available | 19 (65.5%)  5 (17.2%)  1 (3.5%)  0 (0%)  4 (13.8%) |

CSI: craniospinal irradiation; Gy: Gray; CR: complete response; PR: partial response; SD: stable disease; PD: progressive disease
